# Clinical Importance of Molecular Biomarkers in Pleural Mesothelioma

**DOI:** 10.3390/cancers18040679

**Published:** 2026-02-19

**Authors:** Logan Roof, Kenna Koehler, Claire Verschraegen

**Affiliations:** Division of Medical Oncology, Department of Internal Medicine, The Ohio State University Comprehensive Cancer Center, Columbus, OH 43210, USA

**Keywords:** pleural mesothelioma, predictive biomarkers, prognostic biomarkers, immunotherapy, precision medicine

## Abstract

Pleural mesothelioma (PM) is a rare malignancy with opportunities for improvement in current treatment paradigms despite recent advances in systemic therapy. While histology remains the most clinically relevant prognostic indicator, the expanding use of immunotherapy and ongoing clinical trials involving targeted therapies have increased interest in the development of predictive and prognostic biomarkers in this disease. This review summarizes the current biologic and therapeutic landscape of PM and the biomarkers that influence prognosis and treatment response. Biomarkers such as programmed death ligand 1 (PD-L1) expression and tumor mutational burden (TMB) demonstrate inconsistent predictive value in PM and are not currently used in clinical decision pathways in the real-world setting. This review highlights the developing role of dynamic biomarkers such as circulating tumor DNA (ctDNA) for molecular response assessment and minimal residual disease (MRD) detection. This review also examines important genomic and transcriptomic alterations in PM, such as MTAP, BAP1, CDKN2A, and NF2/YAP/TEAD. These alterations provide potential targets for ongoing early-phase clinical trials. Future advances in PM will depend on the development and integration of comprehensive biomarker models that combine clinicopathologic, immune, and molecular features of this complex and heterogenous disease.

## Simple Summary

Pleural mesothelioma is a rare and aggressive cancer involving the lining of the lung, called the pleura, and is often associated with exposure to asbestos. This cancer is difficult to treat, and survival remains poor despite the recent development of new treatments. Current treatments include surgery, radiation, chemotherapy, immunotherapy, targeted therapy, and combinations of these modalities. At this time, it is difficult to know upfront which patients will benefit from which treatments. It is important to develop tools that can predict which patients may benefit from certain treatments and how to tailor these treatments over time. This review discusses the existing data on promising new tools to help guide personalized treatment approaches with the goal of improving outcomes for patients with mesothelioma.

## 1. Introduction

Pleural mesothelioma (PM) is an aggressive, incurable malignancy that arises from the pleural lining of the lung [1]. In the United States (US), PM is newly diagnosed in about 3000 patients per year, with a male predominance and higher risk with older age [2]. The median survival from time of diagnosis is 1 year [3]. Asbestos exposure is the most important risk factor for PM development [4,5]. Widespread use of asbestos peaked in the 20th century, with occupational exposure noted most frequently in the mining, manufacturing, shipping, and insulation industries, as well as in the armed forces [5]. Asbestos use decreased in the 1980s–1990s; however, there was no formal ban on its use in the US until 2024 [6]. PM has a long latency period, with diagnoses often being made 20–40 years after exposure [7]; therefore, it will take many years to understand how the recent asbestos ban will affect the incidence of PM.

PM can present with pleural thickening, pleural nodules, invasion of adjacent organs, and pleural effusions, resulting in symptoms such as dyspnea, pain, cough, weight loss, and fever [8]. The World Health Organization (WHO) divides PM into 3 histologic subtypes: epithelioid, sarcomatoid, and biphasic [9,10]. Histology is the primary prognostic factor in PM [10]. Epithelioid is the most common subtype with the best prognosis, while sarcomatoid is the second most common with the worst prognosis; biphasic contains both epithelioid and sarcomatoid components [9]. The use of platinum and pemetrexed combination chemotherapy regimens has been the standard of care for PM for more than 20 years [11]. Although the US Food and Drug Administration (FDA) approved immunotherapy for PM in 2020, there have not been many additional approved advances in treatment since that time [12,13]. Immunotherapy has a modest effect, with benefit noted mainly in patients with non-epithelioid histologies [14,15].

Our understanding of the genomic landscape and biological pathways in PM is limited. Loss-of-function mutations in *BAP1*, *CDKN2A*, and *NF2* are well-described [3,16,17,18]. Bueno et al. additionally reported mutations in *TP53*, *DDX3X*, *SETD2*, *SF3B1*, and *TRAF7* [16]. Therefore, it is crucial to continue investigating biomarkers in the pursuit of novel and targeted PM treatments.

The goal of this review is to evaluate genomic and transcriptomic alterations in PM and how these affect prognosis and treatment options. Additionally, we evaluate the factors that predict response to PM treatment.

## 2. Current Landscape

### 2.1. Surgery

PM treatment largely depends on stage and histology. Although most patients will not qualify for surgery, clinical stage I (T1, N0, M0) epithelioid PM can be considered for surgical resection (with neoadjuvant or adjuvant systemic therapy), systemic therapy ± pleural intensity modulated radiation therapy (IMRT), or observation [19]. Patients with clinical stage II-IV PM, regardless of histology, have limited local treatment options and should receive systemic therapy [19]. The role of surgery in patients not fitting the above criteria continues to be explored in clinical trials. 

### 2.2. Platinum Doublet Chemotherapy

Combination chemotherapy with a platinum agent (such as cisplatin or carboplatin) and pemetrexed is a frontline PM treatment option. This was FDA approved in 2004 based on the phase 3 study of cisplatin plus pemetrexed vs. cisplatin alone, which showed an improvement in median overall survival (OS) of 12.1 months vs. 9.3 months and progression-free survival (PFS) of 5.7 months vs. 3.9 months, respectively [11,19]. Subsequent studies demonstrated activity of carboplatin with pemetrexed in unresectable PM [19,20,21,22].

### 2.3. Bevacizumab

Bevacizumab, although not FDA approved for the treatment of PM, is commonly utilized in combination with chemotherapy and is included in the National Comprehensive Cancer Network (NCCN) guidelines. The inclusion of bevacizumab in the PM treatment paradigm is based on various trials, each demonstrating approximately 3 months or less of a survival benefit with the addition of bevacizumab to other systemic treatments, such as chemotherapy and immunotherapy [19,23,24,25].

### 2.4. Immunotherapy

Immunotherapy, with or without chemotherapy, is a more recently approved treatment option for unresectable PM. CheckMate 743 was a randomized, phase 3 trial investigating the combination of nivolumab (3 mg/kg once every 2 weeks) plus ipilimumab (1 mg/kg once every 6 weeks) for up to 2 years compared with platinum plus pemetrexed for up to 6 cycles in untreated PM. The trial showed a statistically significant improvement in OS in the immunotherapy group (18.1 months) vs. the chemotherapy group (14.1 months) [13]. The combination of nivolumab plus ipilimumab for first-line PM treatment received FDA approval in 2020. KeyNote-483 was a randomized, phase 3 trial investigating the addition of pembrolizumab (200 mg every 3 weeks) to platinum doublet chemotherapy vs. chemotherapy alone in untreated PM. This trial demonstrated significantly longer median OS in the pembrolizumab arm (17.3 months) compared to chemotherapy alone (16.1 months) [12]. Pembrolizumab subsequently received FDA approval, in combination with chemotherapy, in 2024. Single-agent nivolumab as well as combination nivolumab–ipilimumab are treatment options in previously treated, relapsed PM based on the results of the CONFIRM, INITIATE, and MAPS2 trials [26,27,28]. Interestingly, spontaneous remission of PM has been reported without any treatment, suggesting some autoimmune response to the disease [29,30,31].

## 3. Predictive Biomarkers

### 3.1. Histology

Histology has been shown to be predictive of prognosis and survival in PM; however, it also serves as a predictor of response to certain treatments. Median OS in patients treated with chemotherapy differed significantly between epithelioid (16.5 months) and non-epithelioid (8.8 months) histologies [13]. In a retrospective study of 189 patients treated with first-line chemotherapy, patients with epithelioid histology had improved median OS (26.7 months vs. 15.0 months in non-epithelioid) and PFS (4.8 months vs. 3.6 months in non-epithelioid), regardless of the platinum agent used [32]. In the pivotal phase 3 randomized CheckMate 743 trial, the median OS was improved with combination nivolumab–ipilimumab in the first line compared to chemotherapy, regardless of histology, but a subgroup analysis suggested a greater treatment effect in the non-epithelioid compared with the epithelioid group [13]. Other trials that led to FDA-approved PM treatments have not specifically analyzed treatment response by histologic subgroup. Currently, chemotherapy alone, chemo-immunotherapy combination, and dual immunotherapy regimens are all listed as preferred first-line treatments for epithelioid PM in the NCCN guidelines; however, chemo-immunotherapy and dual immunotherapy regimens are the preferred treatments in sarcomatoid and biphasic histologies based on these results [19].

### 3.2. PD-L1

Programmed death-ligand 1 (PD-L1) expression has been explored in PM as a prognostic and predictive biomarker; however, its clinical utility remains limited. The prevalence of PD-L1 positivity in PM varies widely depending on the assay, cutoff value, and cohort composition, with values reported to range between 20–70%. Expression is noted to be consistently higher in non-epithelioid histologies as compared to epithelioid histology [33,34,35,36]. Tumor heterogeneity in PM further complicates the interpretation of PD-L1 in this disease; several studies have noted discordance between biopsy and resection specimens and between primary and recurrent disease in the same patient. These observations limit the interpretation of results and reproducibility in clinical studies [37,38]. Multiple studies have shown that PD-L1 positivity in PM is associated with worse OS, indicating that PD-L1 is primarily a prognostic rather than predictive factor. This holds true across subtypes, though it has been shown that PD-L1 positivity is more commonly noted in non-epithelioid subtypes and may reflect the association with poorer outcomes [39,40,41].

A flow cytometry analysis on 43 resected PM specimens revealed that PD-L1-positive tumors had more CD45+ leukocytes and higher CD3+ T-cell infiltration, as well as increased proliferation (CD8^+^Ki-67^+^), exhaustion (PD-1^+^TIM-3^+^ CD8^+^), and elevated regulatory T cells and T-cell inhibitory markers compared to PD-L1-negative tumors. These markers were found more often in sarcomatoid and biphasic histologies than in epithelioid. 

However, the predictive value of PD-L1 for response to immunotherapy in PM remains inconsistent across clinical trials. CheckMate 743 included exploratory analyses for PD-L1. While first-line treatment with nivolumab plus ipilimumab improved OS compared with chemotherapy regardless of PD-L1 status, patients with PD-L1 ≥1% demonstrated a numerically higher survival benefit. Some clinical benefit was also seen in those with PD-L1-negative disease. These analyses were not powered to draw statistical conclusions but suggest that PD-L1 is not a particularly reliable predictive biomarker in PM [13,42]. A predictive scoring model was also studied in this trial. A subset analysis utilized a 4-gene inflammatory signature score (measuring the expression of CD8A, STAT1, LAG3, and CD274) that correlated with OS in patients treated with nivolumab plus ipilimumab (median OS 21.8 vs. 16.8 months for high versus low score, respectively) [42]. In single-agent immune checkpoint inhibitor (ICI) studies such as KEYNOTE-028 and KEYNOTE-158, responses were noted in both PD-L1-positive and -negative tumors, and PD-L1 status did not reliably predict clinical responses [26,43,44]. Interestingly, in the CONFIRM randomized study of nivolumab versus placebo in relapsed PM, significantly longer PFS was observed in patients with PD-L1 expression >50% (adjusted hazard ratio for the interaction was 0.28 (95% CI 0.09–0.94, *p* = 0.04) and was not seen for either the PD-L1 >1% or 1–50% subgroups. This finding suggests that PD-L1 may serve as a potential predictive biomarker in those with PD-L1 high disease.

There are several factors that may explain why PD-L1 is not a consistent predictive biomarker in PM. PD-L1 primarily reflects adaptive immune resistance, controlling the initiation and maintenance of immune tolerance and immune evasion within the tumor microenvironment (TME), rather than intrinsic tumor immunogenicity [45]. Numerous studies have shown that the tumor mutational burden (TMB) in PM is relatively low, thereby limiting neoantigen load and subsequent immune recognition [16,46]. Tumor heterogeneity and frequent co-alterations are often noted in PM, contributing to the decreased utility of PD-L1 as a solitary biomarker of response. Of note, deletion of the 9p21 chromosome is frequently found in PM and is associated with a “cold” TME with fewer immune cells, lower PD-L1 expression, and hindrance of immune cell trafficking and activation; this deletion is strongly associated with resistance to anti-PD-1/anti-PD-L1 therapies [47].

Composite biomarker models are likely to better account for the complex interplay between tumor biology and the immune microenvironment, enabling more precise selection of therapy in PM. 

### 3.3. TMB

PM is generally regarded as an immune “cold” tumor with a relatively low TMB. Large genomic profiling studies, including The Cancer Genome Atlas (TCGA) and integrative sequencing projects, have shown that PM has fewer nonsynonymous mutations than other cancer types, with a median TMB often in the range of 1–2 mutations per megabase [16,46]. This likely reflects the underlying biology of mesothelioma, which is driven more by chromosomal losses and tumor suppressor inactivation (such as BAP1, NF2, CDKN2A —see below) than by the accumulation of point mutations [16,46]. As a result, PM samples rarely reach the “high TMB” thresholds used in clinical analyses, limiting the clinical utility of TMB for stratifying patients with PM. 

Several studies have suggested that conventional definitions of TMB, which are largely based on single-nucleotide variants, may underestimate the true molecular landscape in PM. In PM, mutations are often structural variants, chromosomal rearrangements, and larger genomic deletions. Tumor break load and tumor junction burden are metrics aimed at quantifying structural rearrangements and chromosomal rearrangements. These may more accurately capture the molecular landscape in PM [48,49,50]. Additionally, non-canonical neoantigens, such as those derived from gene fusions, aberrant splicing, and other more structural alterations, have been recognized as contributors to tumor immune profiling [51,52]. Tumor break load, tumor junctional burden, and other non-canonical neoantigens may thereby better capture the full neoantigenic potential in PM; they may also predict response to therapies such as ICI and may have relevance for developing novel therapeutic strategies.

### 3.4. ctDNA

Circulating tumor DNA (ctDNA) is an emerging non-invasive method of tumor molecular profiling, monitoring molecular response and minimal residual disease (MRD) and detecting early relapses in certain cancers. There have been several studies demonstrating that ctDNA detection is feasible in PM and that these methods may be used as predictive and prognostic biomarkers of clinical response [53,54]. In a pilot study of a personalized, tumor-informed chromosomal junction assay, seven out of nine patients (four with peritoneal mesothelioma and five with PM) had detectable ctDNA in the plasma (78%) at baseline. The presence or absence of ctDNA after treatment correlated with clinical treatment response, with those having persistent ctDNA noted to have an absence of clinical response and those without ctDNA demonstrating a positive treatment response on imaging [54].

A recent phase 2 study of perioperative nivolumab versus nivolumab plus ipilimumab in resectable PM reported exploratory analyses involving the longitudinal assessment of ultra-sensitive tumor-informed ctDNA assays. The assay detected ctDNA at baseline (C1D1) in 12/26 patients (46%), at C2D1 in 11/25 patients (44%), and at C3D1 in 7/21 patients (33%); 13/25 patients (52%) had detectable ctDNA preoperatively and 2/11 (18%) had detectable ctDNA postoperatively prior to starting adjuvant systemic therapy. This novel type of assay is technically feasible and appears to be clinically relevant in PM. Patients with undetectable ctDNA at C3D1 had significantly longer median PFS: 19.84 months (ctDNA undetectable) vs. 1.41 months (ctDNA detectable) (log-rank test, *p* = 0.027; HR = 0.32, 95% CI: 0.11–0.92). Patients with undetectable ctDNA at the preoperative timepoint had a significantly longer PFS, with a median PFS of 23.26 months (ctDNA undetectable) vs. 2.46 months (ctDNA detectable) (log-rank test, *p* = 0.0059; HR = 0.29, 95% CI: 0.12–0.73) [55]. These findings highlight the potential for ctDNA detection and monitoring in PM with broader, genome-wide tumor-informed strategies in this generally low-shedding/low-TMB tumor type. The future impact of ctDNA in PM is likely to include molecular response assessment during systemic therapy, MRD detection after therapy to refine surveillance approaches, and early relapse detection to enable earlier treatment escalation. 

## 4. Genomic Landscape in PM

### 4.1. MTAP

Deletion of the 9p21 locus is common in PM, frequently involving co-deletion of the adjacent methylthioadenosine phosphorylase (MTAP) gene. Large genomic and fluorescence in situ hybridization (FISH)-based series report MTAP loss in many tumors with homozygous CDKN2A deletion. The overall prevalence of MTAP loss in PM is approximately 40–60%, with higher rates observed in non-epithelioid histologies [16,46].

MTAP functions to break down MTA; when cancers harbor MTAP deletions, this leads to the buildup of MTA, thereby inhibiting another enzyme called protein arginine methyltransferase 5 (PRMT5), which regulates numerous cellular pathways. MTAP-deleted cancers become highly dependent on PRMT5, creating a synthetic lethality by which further suppression of PRMT5 selectively impairs MTAP-deficient cells [56,57].

PRMT5 inhibitors that can selectively target MTAP-deleted cells are being investigated as monotherapy and in combination with other systemic therapies as a PM treatment. One such study is evaluating the oral selective PRMT5 inhibitor TNG908 in patients with MTAP-deleted solid tumors [58]. Another study is investigating the MTA cooperative PRMT5 inhibitor, AZD3470, in patients with MTAP-deleted solid tumors [59]. BMS-986504 is a compound designed to bind to the PRMT5/MTA complex and enhance its inhibitory effect. It is under investigation in several ongoing clinical studies, including evaluation in patients with solid tumors with homozygous MTAP deletion [60].

MTAP deletion can be identified through immunohistochemistry (IHC), FISH, or next-generation sequencing (NGS) copy-number analysis and is considered a reliable marker in mesothelioma [61]. As clinical trial data of these investigational agents emerge, MTAP is likely to become an actionable biomarker in PM.

### 4.2. BAP1

BRCA-associated protein 1 (BAP1), a tumor suppressor expressed in normal mesothelial cells, is a biomarker in PM [62,63]. BAP1 can be lost sporadically in PM, which occurs in approximately half of cases, or can occur in the germline in 5% of cases as part of a syndrome that includes PM, uveal and cutaneous melanoma, and renal cell carcinoma, among others [62,63,64,65,66]. BAP1 is part of the DNA repair pathway and interacts with BRCA1 to enhance tumor suppressor activity [67]. There are currently no therapies to restore the tumor suppressor function of BAP1; however, it is the target of emerging novel treatment approaches in exploiting BAP1’s role in transcriptional regulation and synthetic lethality, including histone deacetylase (HDAC) inhibitors, enhancer of zeste homolog 2 (EZH2) inhibitors, and poly (ADP-ribose) polymerase (PARP) inhibitors [68,69]. One study is investigating APG-115, an oral, highly potent MDM2 inhibitor [70]. Another study is investigating Tulmimetostat DZR123 (CPI-0209), an oral, next-generation EZH2/EZH1 inhibitor. BAP1 regulation of histones raises the possibility of HDAC inhibitors as a treatment option in PM. The phase 3 VANTAGE-014 trial investigated the HDAC inhibitor vorinostat for the second- and third-line treatment of PM and did not show OS improvement compared to placebo; however, there was no selection for BAP1 loss [71]. CUDC-907 is a dual inhibitor of HDAC and PI3K that has shown promising antitumor activity in PM [72]. BAP1 loss has been associated with EZH2 upregulation [68]. A phase 2 trial enrolled 73 patients with BAP1 loss and showed modest activity with a disease control rate of 54% at 12 weeks with the EZH2 inhibitor tazemetostat [73]. Further trials are exploring the synergistic effect of EZH2 inhibitors in combination with FGFR and ATM inhibitors in BAP1-deficient tumors [74,75]. PARP inhibitors have demonstrated efficacy in BAP1 loss preclinical models independent of BRCA mutation status [76]. The phase 2 MiST1 trial showed a disease control rate of 54% at 12 weeks and 23% at 24 weeks in BAP1-deficient patients with relapsed PM treated with 6 cycles of rucaparib [77]. The phase 2 UNITO-001 trial investigated the combination of niraparib plus dostarlimab in homologous recombination repair (HRR)-deficient non-small cell lung cancer (NSCLC) and PM and showed possible antitumor activity in patients with germline BAP1 mutations but not somatic BAP1 mutations [78].

### 4.3. CDKN2A

CDKN2A is frequently mutated in PM tumors, occurring in 40–60% of patients, but can also be found in the germline [79,80]. CDKN2A is a tumor suppressor gene that produces the p14ARF and p16INK4a proteins, which regulate cell cycle and growth [81,82]. CDKN2A mutation resulting in loss of these proteins leads to unchecked cell growth and survival. Several clinical trials have investigated the efficacy of cyclin-dependent kinase 4 and 6 (CDK 4/6) inhibitors in PM, given the high rates of homozygous CDKN2A mutations in this population. NCT02187783 showed activity of ribociclib in solid tumors with CDK 4/6 pathway alterations [83]. The phase 2 MiST2 trial showed promising clinical activity with a response rate of 54% at 12 weeks in p16INK4A-deficient patients with PM who were treated with abemaciclib [84]. A phase 1 trial (NCT05538572) investigating the novel CDK 4/6 inhibitor PRT3645 in solid tumors showed favorable safety with ongoing dose escalations [85].

### 4.4. NF2/YAP/TEAD

Loss of function of the neurofibromatosis type 2 (NF2) tumor suppressor gene occurs in approximately 30–50% of PM cases. Loss of NF2 is strongly associated with uncontrolled activation of Hippo-Yes-associated protein (YAP) signaling, thereby allowing unphosphorylated YAP to accumulate in the cell nucleus, binding TEA domain (TEAD) transcription factors. These TEAD transcription factors control the expression of genes that promote proliferation, survival, invasion, and immune modulatory effects. Preclinical studies have demonstrated that NF2-deficient PM models are dependent upon YAP/TEAD, making this a potentially targetable pathway for drug development [16,46,86,87].

There is a current phase 1 study investigating the oral TEAD inhibitor VT3989 in patients with PM and other solid malignancies with and without NF2 mutations (NCT04665206). Results from the dose escalation phase 1 study showed encouraging tolerability and durability of responses in patients with PM. The dose expansion and combination portions of the study are ongoing [88]. Another first-in-human phase 1 study is examining the role of the pan-TEAD inhibitor ISM6331 in patients with advanced or metastatic PM or other solid tumors, with preliminary clinical results pending [89].

NF2 status is not yet used to guide standard therapy in clinical practice, but it has emerging relevance for clinical trial enrollment and treatment selection. NF2 and YAP/TEAD pathway activation may serve as future predictive biomarkers for targeted therapy and prognostic biomarkers for clinical response and outcomes. 

These biomarkers and their clinical significance are outlined in Table 1. Table 2 lists the ongoing clinical trials utilizing these recognized molecular clinical biomarkers. 

### 4.5. Tumor Microenvironment

PM has a low TMB and a microenvironment composed of anergic and immunosuppressive cells [90]. Macrophages, natural killer (NK) cells, and T cells make up the majority of cells in the TME and are thought to promote an inflammatory response that promotes angiogenesis and tumor growth [90,91]. Tumor-associated macrophages (TAMs) are a major driver of the inflammatory response that suppresses immune surveillance and promotes tumor growth [92]. One study showed that TAMs had an immunosuppressive effect on monocytes, promoting mesothelial cell proliferation and decreased sensitivity to chemotherapy [93]. Although immune effector cells are present in the TME, studies have shown that T-regulatory cells, especially CD25-expressing cells, also play a role in suppressing immune response to a tumor [91]. Depleting these cells of CD25 reduced tumor growth and improved survival in mouse models [91]. PM cells produce cytokines that further inhibit immune response, such as IL-1, IL-6, IL-10, VEG-F, and TNF-β [91,94]. Additionally, PM biopsies were shown to be deficient in dendritic cells, which are antigen-presenting cells that are crucial for activating effector T cells against the tumor [91,95]. PD-L1 expression is variable in PM, with sarcomatoid and biphasic subtypes having higher expression than epithelioid. However, there is significant variability in the level of T-cell infiltration between PD-L1 high and low tumors, suggesting that PD-L1 expression alone is insufficient to predict response to immunotherapy, and there is likely a more complex interplay between the presence of various immune cells and cytokines in the TME. Beyond PD-L1, studies are investigating other immune checkpoint molecules such as TIM-3, LAG-3, TIGIT, and VISTA as potential treatment targets. Chimeric antigen receptor (CAR) T-cell therapy serves as a possible mechanism to bypass suppressed immune cells by directing the CAR-T cell to targets on the mesothelial cell surface [96]. Additional strategies aimed at altering the TME—increasing dendritic cell differentiation [97], targeting pro-inflammatory cells, and re-educating immune effector cells—serve as possible novel therapeutic targets in PM.

### 4.6. Transcriptomic Landscape

The transcriptomic landscape is an emerging field of interest in PM, where several studies have investigated miRNAs as potential biomarkers. Kirschner et al. [98] found that miR-29c and miR-92a were elevated in plasma samples and miR-625-3p was elevated in tumor specimens in PM patients. Zhu et al. [99] reported elevated levels of miR-19b, miR-26a, miR-26b, and miR-29a in PM tumor tissue, as well as significantly elevated levels of miR-19b and miR-29a in the plasma of PM patients when compared to control patients with lung cancer, asbestos exposure, and healthy controls. Balatti et al. [100] reported upregulated levels of miRNAs in the miR 17-92 cluster in PM tissues. These miRNAs, among many others that have been studied, as outlined in two reviews by Martinez-Rivera et al. and Tonnini et al. [101,102], serve as possible diagnostic biomarkers for PM. There is also interest in the prognostic capability of miRNA in PM. One study showed that elevated level of hsa-miR-98 was significantly associated with poor prognosis in PM patients with previous asbestos exposure [103]. The Mesothelioma Avastin Cisplatin Pemetrexed Study (MAPS) was a phase III trial that investigated the impact of angiogenesis-related microRNAs on survival in PM patients. This study found that low miR-193b-3p expression was associated with prolonged survival in PM patients. Additionally, the investigators established that low expression of miR-155–5p, miR-29c-5p, miR-132–3p, and miR-100–5p was associated with increased survival in the bevacizumab plus chemotherapy arm [104]. There are several ongoing clinical trials further investigating miRNA in PM [105,106,107], demonstrating the promising role that miRNAs could serve in the diagnosis, prognosis, and potentially treatment selection and response in PM patients.

**Table 2:** Ongoing Clinical Trials.

Molecular Marker	Investigational Drug	Clinical Trial Information
MTAP	TNG908: oral selective PRMT5 inhibitor	Safety and Tolerability of TNG908 in Patients With MTAP-deleted Solid Tumors NCT05275478
MTAP	AZD3470: oral, second-generation MTA-selective PRMT5 inhibitor	A Study of AZD3470, a PRMT5 Inhibitor, in Patients With MTAP Deficient Advanced/ Metastatic Solid Tumors (PRIMROSE) NCT06130553
MTAP	BMS-986504: oral MTA cooperative PRMT5 inhibitor	A Study to Evaluate the Mass Balance, Metabolism, Elimination, and Drug Levels of [14C]-BMS-986504 (MRTX1719) in Participants With Advanced Solid Tumors With Homozygous Methylthioadenosine Phosphorylase Deletion NCT06672523
BAP1	APG-115: oral, highly potent, MDM2 inhibitor	Alrizomadlin (APG-115) in Subjects With BAP1 Cancer Syndrome and Early-Stage Mesothelioma NCT06654050
BAP1	DZR123: dual EZH2/EZH1 inhibitor	A Study of Tulmimetostat DZR123 (CPI-0209) in Patients With Advanced Solid Tumors and Lymphomas NCT04104776
TEAD	VT3989: oral TEAD inhibitor	Study to Evaluate VT3989 in Patients With Metastatic Solid Tumors NCT04665206
TEAD	ISM6331: pan-TEAD inhibitor	Study of ISM6331 in Participants With Advanced/ Metastatic Malignant Mesothelioma or Other Solid Tumors NCT06566079

## 5. Discussion

These studies highlight the progress and ongoing limitations of biomarker-driven treatment strategies in PM. Histology remains the most clinically relevant factor when informing prognosis and stratifying systemic therapies, with immunotherapy solidified as a standard front-line treatment in non-epithelioid histologies. PD-L1 and TMB can be helpful to characterize tumors but have shown inconsistent predictive value in this setting. These limitations highlight the need for more comprehensive assays, such as the exploratory biomarker analysis of a 4-gene inflammatory expression signature score conducted as part of the phase 3 CheckMate 743 study. 

There is growing evidence that ctDNA analysis may play a role in the management of PM, guiding escalation and de-escalation strategies for systemic therapy. Tumor-informed ctDNA assays have demonstrated more clinical utility in PM, as PM tumors have been shown to have a relatively low percentage of shedding in preclinical studies. 

Genomic and transcriptomic analyses have provided more robust insights into the underlying biology of PM and possible therapeutic targets. Alterations in MTAP, BAP1, CDKN2A, and NF2 are commonly found in PM and may help to identify unique molecular vulnerabilities for therapeutic intervention. There are many ongoing early-phase clinical trials investigating potential targeted therapy combinations in PM. These studies are promising and indicate the possibility of a shift toward biomarker-driven PM treatment and precision medicine in the future. 

Currently, predictive and prognostic biomarker-driven treatment in PM remains limited in clinical practice. Important limitations of the present landscape of biomarker-driven care in PM are the variability in strength and maturity of the supporting evidence. Much of the available literature consists of retrospective analyses of tumor specimens, small translational cohorts, and exploratory biomarker analyses within early-phase clinical studies. For example, PD-L1 expression and inflammatory gene signatures have been explored through post hoc and exploratory analyses, rather than through prospectively designed biomarker studies. Many genomic biomarkers, including MTAP loss, NF2/YAP/TEAD alterations, and BAP1 loss, have been evaluated in preclinical studies and early-phase clinical trials. Key gaps in the field include the lack of prospectively validated predictive models, limited standardization of assays due to the underlying heterogeneity and biological complexity of PM, and underutilization of biomarker-driven and biomarker-stratified designs in larger phase III clinical trials. Although difficult to study in this rare disease, future progress will depend upon the development of standardized assays, prospective validation of these predictive and prognostic biomarkers, and the integration of prespecified biomarker endpoints in late-phase clinical trials. Comprehensive models incorporating clinicopathologic features, immune signatures, genomic alterations, and biomarkers will offer the best opportunity for implementation into clinical practice and the individualized treatment of patients with PM.

## 6. Conclusions

While PM has received public attention with litigation against asbestos companies, much needs to be learned about the biological pathway aberrancies induced by asbestos. We have described the known evidence and clinical trials that are testing potential therapies for this disease. Because PM remains rare, it is difficult to gather enough tumor specimens to further explore the relevance of known biological pathways and to discover new pathways that can be targeted therapeutically. Currently, some known factors help predict survival, but none have been shown to prolong survival in a meaningful way. Immunotherapy has brought the latest, albeit modest, advances in this disease. A deeper understanding of the relationship between the immune system and mesothelioma is needed to improve outcomes. We encourage the oncology community to collaborate in a cohesive fashion to improve the prognosis of our patients diagnosed with PM.

## Figures and Tables

**Table 1:** Biomarkers in Pleural Mesothelioma.

Biomarker	Biological Role	Key Studies	Setting	Clinical Use
PD-L1	Immune checkpoint ligand; marker of immune activation and suppression	CheckMate 743; KEYNOTE-028; KEYNOTE-158; CONFIRM	First-line (CM743); Later-line (KEYNOTE-028/158, CONFIRM)	Exploratory predictive/prognostic biomarker; some data to suggest possible predictor of response in PD-L1 <50%
TMB	Surrogate of neoantigen load	CheckMate 743 exploratory analyses; KEYNOTE-158 mesothelioma cohort; Samstein et al. pan-cancer	Various ICI trials	Exploratory biomarker; no robust predictive signal due to low TMB
ctDNA	Tumor-derived DNA in plasma for MRD and response monitoring	Perioperative nivolumab ± ipilimumab trial (Reuss et al.); Personalized junction assays (Parikh et al.)	Perioperative; post-surgical monitoring	Exploratory biomarker; feasibility of detection; molecular response and MRD
BAP1	Chromatin regulation, DNA repair	TCGA/Hmeljak et al.; retrospective ICI studies	Translational	Associated with immune-inflamed phenotypes; exploratory predictor of ICI sensitivity
CDKN2A	Cell-cycle control	TCGA; immunogenomic outcome cohorts; retrospective ICI studies	Translational; retrospective studies	Prognostic; associated with immune-cold states and poor ICI outcomes
MTAP	Polyamine metabolism; synthetic lethality with PRMT5/MAT2A	IDE397; TNG908; AZD3470; BMS-986504/MRTX1719	Early-phase targeted therapy trials	Predictive biomarker for MAT2A and MTA-cooperative PRMT5 inhibitors
NF2/TAP/TEAD	Hippo pathway regulator	TCGA; Hmeljak et al.; VT3989; AZD6284	Translational; early-phase targeted therapy trials	Predictive biomarker for YAP/TEAD pathway targetable dependence

## Data Availability

No new data were created or analyzed in this study. Data sharing is not applicable to this article.
